# Online Newspaper Framing of Non-Communicable Diseases: Comparison of Mainland China, Taiwan, Hong Kong and Macao

**DOI:** 10.3390/ijerph17155593

**Published:** 2020-08-03

**Authors:** Angela Chang, Peter J. Schulz, Angus Wenghin Cheong

**Affiliations:** 1 Department of Communication, Faculty of Social Sciences, University of Macau, Macao, China; 2 Institute of Communication and Health, Lugano University, 6900 Lugano, Switzerland; peter.schulz@usi.ch; 3 ERS e-Research & Solutions, Macao, China; angus@e-research-lab.net

**Keywords:** risk factors, framing, causal assertions, non-communicable disease, online press, behavioral factor

## Abstract

As non-communicable diseases (NCDs) are now well recognized as the leading cause of mortality among adult populations worldwide, they are also increasingly the focus of media coverage. As such, the objective of this study is to describe the framing of NCDs in the coverage of newspapers, with the understanding that it says something about the society producing it. Automatic content analysis was employed to examine disease topics, risks, and cost consequences, thus providing lay people with a chance of learning the etiology of NCDs and information available for fighting diseases. The result of the computational method identified a total of 152,810 news articles with one of the seven supra-categories of NCDs. The category of metabolic diseases was covered most frequently in the past ten years. Three health risks received ample attention in all 11 newspapers: stress burden, tobacco use, and genetic predispositions. The results evidenced how media framed risk information of illnesses would distort the way in which diseases were selected, interpreted, and the outcome communicated. Future research building on our findings can further examine whether news framing affects the way the readers perceive and prevent NCDs.

## 1. Introduction

Non-communicable diseases (NCDs) are the result of a combination of genetic, physiological, environmental and behavioral factors as reported by the World Health Organization (WHO) [1]. Previous studies indicated that five NCDs—cardiovascular diseases, chronic respiratory diseases, cancer, diabetes, and stroke—make up the lion‘s share of causes of deaths worldwide; four health-relevant behaviours are crucially important as risk factors: unhealthy diet, lack of exercise, tobacco smoking, and alcohol consumption [1,2,3,4]. Therefore, it is generally understood that reduction of these behaviours would result in a considerable decrease in the prevalence of the major NCDs.

The Chinese population is experiencing a rapid increase in the prevalence of NCDs. The WHO report indicated that NCDs accounted for 8,577,000 deaths (85–87% of total deaths) in Mainland China [5,6]. Cardiovascular diseases, cancers, and chronic respiratory diseases are the major causes of death of NCDs [6]. The top two behavioral risk factors of NCDs for Chinese adults are tobacco smoking and alcohol consumption, while the two most dangerous illnesses are hypertension and obesity [7]. More recent epidemiological evidence also indicated that smoking ranked as the leading risk factor for men, while metabolic risk factors affect disability-adjusted life years in women in China [8]. To be specific, the estimate of the prevalence of metabolic syndrome among adults in Mainland China was 24.5%, which was higher than the worldwide prevalence of 20–25% [9].

NCDs are a grave danger not only in mainland China, but also in the neighboring territories that have long been part of Chinese culture: Taiwan, Hong Kong, and Macao. In Taiwan, diseases such as cardiovascular related, cancer, and diabetes are listed in the top causes of deaths of NCDs, accounted for 110,720 or 77% of deaths [10,11]. Cancer and diabetes remain the top two causes of premature mortality in Taiwan and diet-related obesity has also increased steadily in adults since 1980 [8,12]. In Hong Kong (HK), NCDs accounted for 35,365 deaths in 2015 (77% of total HK deaths) [13]; neoplasms and the disease of circulatory and respiratory system together accounted for about 78% of all deaths. Concomitantly, NCDs have been the leading cause of death in Macao since the 1970’s [14]. The current profile reveals that NCDs account for approximately 76% of total deaths in Macao; specifically, cancer, cardiovascular diseases, and lung disease account for nearly 60% of all deaths in the past years [15]. Macao’s overall cancer statistics draw a picture very similar to HK and the other metropolitan cities, notably Shanghai and Beijing in Mainland China. Occurrence of the different types of cancer (i.e., colon and rectum) and the ranking order for cancers in female adults were very similar in Macao and HK [13,14,15], due to the similar socio-economic conditions of these two areas.

Studies have concluded that a healthier diet and more physical activity would reduce the prevalence of NCDs for Chinese, which have been spurred by economic growth, urbanization, and a rapid spread of westernized diet [16,17]. Thus, the dual purpose of this study is to understand how and which NCDs are covered in mainstream news environment and what their content says about risk assessment and cost consequences for Chinese speaking readers. The analysis consists of comparisons between mainland China and its three neighboring territories, Taiwan, HK, and Macao. The three are situated, geographically and historically, within the Chinese civilization. Due to political events and developments, they were set on different political paths at the time of European colonization (HK and Macao) or later, as part of the ideological wars of the 20th century (Taiwan). As such, the recent development and change in media landscape of the three territories was quite different from mainland China and also different from each other.

As behavioral factors play an important role in the etiology of NCDs and consequently in the prevention of these conditions, information provided by news is an important resource in fighting the burden of NCDs. This makes a built environment of news coverage of these conditions in general and the risk assessment in particular important factors in the endeavor to move against the damages done by NCDs. Recognizing the need for strategic health communication surrounding the causes and implications of disease ought to be a main task of advocates from media. We begin by outlining the theoretical approach and application of the automatic method to be followed throughout the paper. 

### Health and Risks Coverage

When examining how the media present health topics, researchers have replied on the theoretical construct of framing, which refers to the channels and content used to describe issues [18,19,20]. Numerous viewpoints from the high-income countries extend framing analysis of many kinds of themes to develop a framing typology of disease risks; it includes frames of medical, behavioral, or structurally focused; gain vs. loss frames; risk-amplifying vs. risk-attenuating frames; or episodic vs. thematic frames [21,22,23,24,25].

The frame of episodic or thematic is one of the most widely used frames for examining attribution of health risks and costs in health communication study; literature on public health shows that American news serves as a major source in highlighting individual’s risks by episodic framing while it decreases adverse outcome for society by thematic framing [26,27,28]. The pattern of American media is observed to blame individuals for their own health afflictions, but with little consideration of larger societal and political claims of systemic causation [18,29,30]. Namely, the thematic frame lacks news attention for social forces and governmental responsibility for solutions [31]. Similar findings were also observed in the Chinese news by emphasizing more episodic frame of risk factors than thematic consequences on certain diseases or mental disorder [32,33,34,35]. Consistent with individual and societal environmental framing studies, researchers evidenced that defining a health problem in individual terms potentially limits social and governmental responsibility for addressing it. In contrast, framing by population-level structural determinants assumes that knowledge of NCDs would motivate people to broaden their focus by demanding action from business, government, and larger social forces to solve the problem. 

Framing has been used extensively in news content research, which holds that the way media present a particular frame affects the way the public thinks about it [31,36]. The framing hypothesis in journalism indicates that surrounding the issue can change the reader’s perception without having to alter the actual facts as the same information is used as a base. For instance, framing of medical-scientific progress related to health risks in the practices of leading press outlets is advised to better to educate lay people [37,38]. It is done through the journalists’ choice of certain topics, words, or images to cover a news story. However, a different view showed that news media may not exert significant influence in the formation of risk perception; news agenda followed a layman’s perspective of risk instead of reflecting the perspective of scientists or governments [39]. Hence, several studies support that the combining use of frames and agenda would mitigate the negative image of an incidence for patients, caregivers, and health science professionals [29,30,40,41,42].

In sum, studies suggested that journalists’ or editors’ framing typology of certain diseases undermines readers’ understanding of health problems; it may also lead to lack of choices for considering societal or governmental responsibility in a news built environment [19,20,22]. Previous studies suggest that framing individual’s risk factor tends to invoke individuals receiving blame while weakening support for broader consideration from governmental or environmental perspectives. Thus, adopting more thematic framing by citing incidence levels and population statistics can increase support for the societal solutions and governmental support. The framing of NCDs as caused either individually or socially is likely to prefigure the challenge these conditions put to government [18,40,43]. As attribution of the detrimental behaviors to societal forces becomes dominant, the challenge for government to move against these behaviors grows. A government willing and able to fight NCDs might welcome this framing, while a government unwilling to do it or incapable of doing it, might dislike this framing. 

Comparing mainland China and the three territories, in addition to the research questions, provides evidence to be interpreted in light of the framing considerations. Based on all this, four research questions (RQ) are raised to learn what dominant media coverage reveals about convergence of framing and agendas of NCDs in news discourse:

(a) How much coverage was devoted to NCDs in mainland China newspapers, and in the neighboring areas, and how did that change over time?
(b) How were the NCDs covered along with risk assessment?
(c) How were the NCDs covered along with cost consequences?
(d) What associations, if any, do Chinese news attribute risks with individual-level (episodic theme) or social-level frames (thematic theme)?

## 2. Materials and Methods

Lay people usually seek health information from a range of sources, including the newspaper, which serves as an important source of information [25,33,44]. In this regard, newspapers are well recognized as one of the most utilized sources of health information in Taiwan [45] and China [46]. Given the traditional sensationalism of health subjects in magazines, the brevity and the volatility of contributions in radio and television, and the unsolved quality issues of health webpages, the newspaper (including online) can still be considered the most reliable medium for distributing health information. Hence, online newspapers have become important and credible sources of health information, allowing readers to access unlimited information and understand more about disease prevention [22,47]. 

Content analysis has been one of the most important methods for studying media discourse [36,37]. Recently, the computational method of automated content analysis has become an important tool in advancing metadata collection and analysis from interdisciplinary perspective [48,49,50,51,52,53]. In our view, this research has two assets: (1) one of the first large-scale of comparative analyses of online press in Chinese besides English, and within different politically placed Chinese populations (mainland China, HK, Taiwan, Macao); and (2) a methodology of automated analysis utilizing computing facilities for collecting and parsing text material for large online population of Chinese readers over a decade. 

### 2.1. Newspaper and Article Sampling 

The sampling of mainland China newspapers followed previous studies, which included Chinese-language, top circulation of daily news, and accessible via flagship online websites of the newspapers [35,54]. Additionally, national newspapers and regional newspapers from the most populated cities with severe NCD problems were also considered [5]. A total of four newspapers run either by market-driven organizations or central government from mainland China were selected: People’s Daily News (人民日報), Southern Metropolis Daily News (南方都市報), Guangzhou Daily News (廣州日報), and Beijing Evening News (北京晚报). 

For the areas outside mainland China, the selection criteria were as similar as possible. The process included the flagship websites of newspapers allowing people from all over the world to read, free access to full version, and providing up-to-date news. As such, three most highly circulated newspapers were selected in Taiwan: United Daily News (聯合報), Times Daily News (中國時報), and Liberty Times (自由時報). In HK, three mainstream newspapers with claimed daily readership of millions were selected: Apple’s Daily News (蘋果日報), Oriental Daily (東方日報), and MingPao (明報). In Macao, one oldest and largest newspaper, Macao Daily News (澳門日報), subsidized from the government was selected. The e-paper versions of sampled newspapers are usually uploaded from the whole printed version for promoting online readership. 

To locate articles, search terms as target words were chosen from categories of NCDs for computational procedure [5]. Any article that contained one or more of 15 target words related to NCDs—Chinese names of diseases and risk factors as important causes of diseases—was selected. Considering differences between nationwide standard of Mandarin and localized languages in terms of phrase usage in formal journalism writing, special attention was noted in the codebook. Articles were screened for sampling if they contained any one of a list of target words at least twice to ensure relevance and accuracy. Appendix A lists the 15 target words of diseases along with key risk assessment in both English and Chinese. 

This study documents the content of news stories that appeared between 1 January 2010–31 December 2019. The sampling procedure can be considered to yield a representative newspaper story of NCDs, rather than the statistical sense of the keyword. Various aspects have to be considered such as removing articles that did not meet the inclusion criteria of a news-type to come to this conclusion. Sampling of newspapers was as similar as could be and aimed at the leading organs, which have long been recognized as formative for the followers with regard to style and issue agenda. There was no further sampling among the articles produced by the search terms; with regard to article selection, the representativeness is therefore a lesser issue. In terms of subjects (NCDs), the most prevalent diseases were chosen for inclusion in the study, thus capturing the medical conditions that are important from a public-health perspective. In terms of content, the categories of the illnesses is a pertinent search term, which was supplemented by several specific terms of diseases. 

### 2.2. Codebook Development 

A codebook was developed including 26 specific diseases under seven disease categories, 13 risk assessments, and eight consequences of NCDs by referring to previous studies [2,3,7,14,55]. The codebook developed in this study was not only based on the demands of the research question but also on the methods available, the nature of the medium under scrutiny, and its content. We aimed to analyze online press, and the codebook design needed to remain open to adjustments before, during and after the coding process. Therefore, a pilot test enabled the mapping of internet-specific news that could be automatically coded by computer and equally identified the NCDs related text by manual checking were imperative [34,50,53,56]. Hence, the coding procedure captured explicit assertions as much as possible, despite not exhaustive selection. The disease categories, risk assessments, and consequences of NCDs for the codebook are displayed in the Appendix B for the searched terms in Chinese and then translated into English.

### 2.3. Machine Coding Process 

News data collection began with customized data storage, selection, and screening. It was done with a tailored software, DivoMiner, a tool for automated content analysis by e-Research & Solutions (e-RS) in Macao. An exploratory test was run several times. In addition, a test for validity and reliability of the machine work was run by confronting computer coding with human notations. A randomly assigned 1% of the sample articles were examined by the first author and four trained research assistants. An acceptable level of agreement of 80% was reached at the end. A substantially acceptable level of the interrater reliability for the raters was found to be substantial Cohen’s Kappa = 0.78 (*p* < 0.001), 95% CI (0.604, 0.948). This signifies an appropriate method for selecting material and guarantees data quality. 

## 3. Results

**Figure 1:**
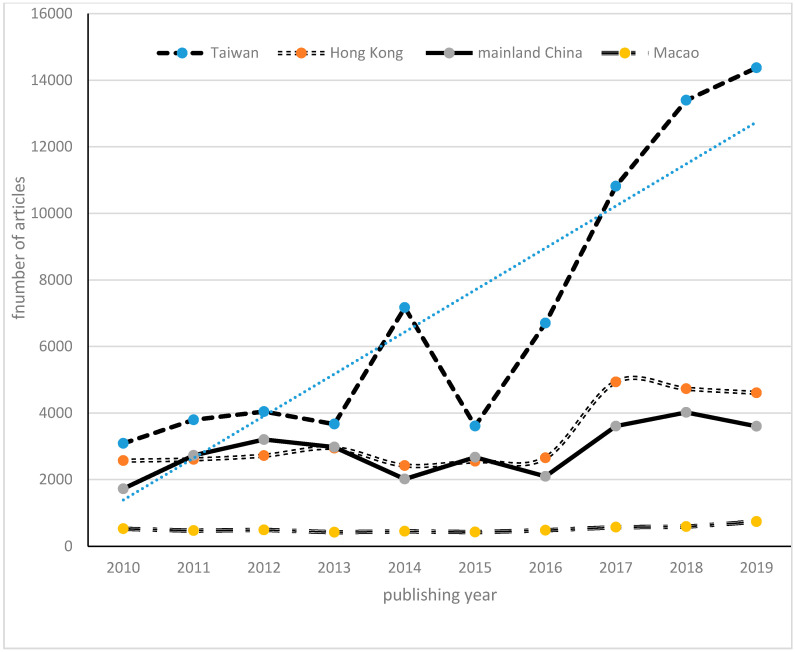
Number of non-communicable disease (NCD)-related articles in Chinese newspapers in mainland China and three neighboring areas, 2010–2019.

A total of 137,175 newspaper articles covering NCD, risks, and consequences of diseases were identified, corresponding to an average of 13,715 stories every year. The trend line over the 10 years shows a gradual increase in all regions. It is because three Taiwan newspapers together mirrored the overall development most closely. A clear trend towards overall attention of NCDs starts in 2017, and the overall attention declined to 9251 articles in 2015. NCD-related coverage in China shows comparatively low coverage in 1721 stories in 2010 and in 2014 stories in 2014. There was much of a resurging interest of NCDs coverage in all areas between the years of 2017 and 2019. The attention to NCDs in Macao, in comparison, remains very stable and does not display much change over time. Figure 1 shows the developments of NCDs coverage in Mainland China, Taiwan, HK and Macao for ten years in overview. 

A total amount of 152,810 articles collected explicitly addressed at least one of the seven supra-categories of illnesses, among 137,175 initially collected articles. To be specific, the coverage of metabolic diseases (40.8%) in mainland China widely outnumbered other disease categories, comparing with cancer (19.9%), autoimmune syndrome (18.1%), and cardiovascular disease (16.6%). In the neighboring areas, the coverage of metabolic diseases was also paid the most attention, followed by cardiovascular diseases. In contrast, three disease categories (i.e., musculoskeletal, neurological decline, and chronic respiratory) were the least covered. Newspapers in these four areas differed significantly in the attention paid to them ($x^{2}$ = 1870.71, df = 28, *p* < 0.001). Table 1 displays the comparison of the coverage of disease categories in mainland China and in the neighboring areas. 

The total amount of 46,586 (33.96%) articles contained at least one risk assessment of NCDs, among 137,175 articles initially collected. Overall, the top three causal agents such as stress burden, genetic predispositions, and a tobacco use received more journalists’ attention in all regions. Specifically, there was a substantially stronger focus on risk factors of “stress burden” (range from 25.1–29.9%), genetic predispositions (range from 18.6% to 26.3%), and tobacco use (range from 13.6–19.6%) than other causes of NCDs. In contrast, three risk factors such as social and economic system, poor diet, and personal and family traits were least covered (range from 0.0–0.3%). Notably, a risk factor of personality and psychological predispositions had only three stories covered in three newspapers. Although a similar distribution of risk factors was found, the numbers and ranks of NCDs’ risks were significantly different in mainland China and the neighboring areas ($x^{2}$ = 1881.95, df = 36, *p* < 0.001). Table 2 displays the risk assessment of NCDs in mainland China and the neighboring areas for the past 10 years.

Taking all newspapers together, NCD costs were merely presented in a total of 2272 (1.66%) articles addressing at least one explicit consequence of illnesses, among the total amount of 137,175 articles collected. Overall, the main consequence such as societal costs outnumbered the other attributes (range from 37.0–71.6%). In comparison, the cost consequence such as employer costs received least journalist’s attention (range from 0.0–1.6%). Another two cost consequences such as other diseases (range from 0.3–6.0%) and loss quality of life (range from 0.0–13.0%) also received low values of coverage. A similar trend of cost consequences was observed in Taiwan and HK, however, the numbers and ranks of NCDs’ consequences were significantly different among these areas ($x^{2}$ = 223.39, df = 21, *p* < 0.001). Table 3 displays the cost consequences of NCDs covered in newspapers from mainland China and the neighboring areas.

A total of eight frames of individual-level risks (episodic theme) (i.e., poor diet, lack of exercise, consumption, tobacco use, drugs other than alcohol or tobacco, stress burden, personal and family situation, and personality traits) were observed at lion share (81.7%), compared with four social-level of frames (thematic theme) (i.e., pollution, poverty, social and economic system, and genetic predisposition) (18.3%). It is interesting to note that the thematic themes were more prevalent in framing on social costs (79.3%), while the episodic themes on individual-level costs were relatively low (20.7%).

The cluster analysis was further conducted for graphical representation of the hierarchical tree in order to verify if a classification of NCDs news was spread through the renowned online press. The risk assessment and casual inferences of NCDs were grouped into four clusters by integrating diseases covered. The different clusters are marked as A, B, C, and D. The *x*-axis means rescaled distance cluster while *y*-axis means the variables measured.

The result of the hierarchical relationship is illustrated in a dendogram in which there are four clusters with the characteristic 26 NCD compounds as variables. One cluster (A) combines three categories of NCDs, six individual (episodic) frame of risks, two social (thematic) frame of risks, two individual costs, and one social cost consequence. A second cluster (B) combines two categories of diseases and one genetic predisposition risk of frame. A third cluster (C) groups two categories of NCDs, two episodic frame of individual’s risk, one episodic frame of individual’s cost, and two consequences of social costs. A fourth cluster (D) combines one disease and one thematic cost frame of social costs. The cluster D integrated metabolic disease and societal cost performing better than the others in simply disclosing news discourse. 

**Figure 2:**
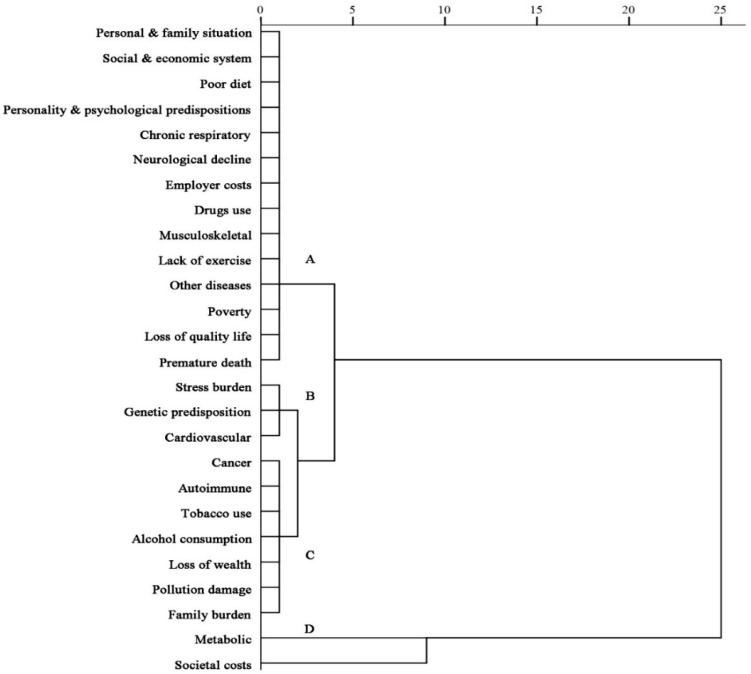
A dendrogram from the hierarchical clusters analysis displays coverage relationship among four clusters of framing NCDs.

In the dendrogram as shown in Figure 2, cardiovascular diseases (e.g., stroke, heart attach), stress burden, and genetic predisposition were grouped in the same cluster B, which suggested that they were closely related. In addition, tobacco use, alcohol consumption, and pollution damage were grouped in the same cluster C, confirming their similarity in coverage. By contrast, societal costs was the only consequence in separated cluster D. The width of the dendrogram indicated the order in which the clusters were joined. A more informative dendrogram was created to show the close association where the widths reflect the distance between the clusters B and C. Interestingly, the dendrogram displays a big difference of coverage between cluster A and that of cluster D. Overall, a dendogram provides a richer context of NCDs agenda in interpreting associations between disease category, risks, and consequence inferences.

## 4. Discussion

The frequency of NCDs coverage on average was observed to be the highest in newspapers in Taiwan and lowest in Macao. The overall trend in this study indicates that general coverage of NCDs in 11 Chinese newspapers has soared since 2017 but that development was almost exclusively created by Taiwan’s newspapers and also by two regional newspapers in mainland China. Consistent with presenting news values on disease associated with framing risk factors from the individual’s perspective, the results suggest that newspapers overall amplified metabolic diseases by emphasizing individual behavioral risk factors. NCD issues in Chinese news do not automatically designate themselves as priority or significant issues with more coverage but rather as issues that are selectively and consciously advanced by journalists and editors. 

Nevertheless, despite the contrast of NCD news amplification of certain diseases, it should be noted several conditions such as neurological decline (i.e., Alzheimer, Parkinson, and multiple sclerosis) were much neglected by all Chinese media studied. Another example is that the category of chronic respiratory disease received the least attention from news media in China (0.2%), Taiwan (0.1%), and HK (0.0%), albeit chronic respiratory disease was considered as one of the top five cause of death for Chinese [2,5,6,13,40]. Furthermore, the built news environment had low values of coverage on two important risks of individual’s behavior, such as poor diets and lack of exercise which are crucially emphasized by the local officials, governments and public health researchers [2,3,13,14]. This result suggests that the lack of media coverage on certain diseases might decrease public awareness and frustrate the caregivers and public health professionals who make efforts in communicating ways of prevention of these conditions [27].

Although People’s Daily News provides direct information on the viewpoints of central government, this official newspaper of the communist party in mainland China was observed to have the least coverage of NCDs and its risk assessment. There was not even an increase of NCD coverage from 2014–2015 found in People’s Daily News to echo China’s national guideline for NCD prevention and treatment [9]. In comparison, regional newspapers such as Guangzhou Daily News and Beijing Evening News increased NCD coverage considerably during 2012–2015. This reflected their shifting of editorial and news emphasis away from chronic conditions to treatment. These attempts at framing the risks and consequences of NCDs in the media can potentially affect individual attitudes, as well as public policy deliberations [40]. Alternatively, media can also omit disease information and risks or remove consequences and solutions from the coverage, which will create gaps in what a large public thinks and knows about the diseases. 

While articles in China seemed to be incongruent with reality in covering major causes of NCDs with regards to content, they exhibited a stronger emphasis on framing of risk factors for metabolic disease. The typified disproportion of NCDs coverage formed news waves by showing that the NCDs coverage did not keep pace with ever-expanding disease burden with adverse consequences [6,8]. In other words, the changing intensity of NCD news reporting reflected the lack of resources in the news media to educate populations and protect the lay people from NCDs. It is evidenced that three risk assessments (i.e., stress, genetic predispositions, and tobacco) received dominant coverage in the Greater China area, although it was unexpected that the factor of stress burden received so much attention in all newspapers. This is consistent with existing studies in depicting the construct of NCD news on risk as caused by individual premises [27,29,30,31,43]; Chinese news also tended to blame individuals for their own health afflictions, rather than considering societal contributions in allocating responsibility for solutions. As NCDs are rooted in the social determinants of health, they cannot be stopped through individual action alone. 

The findings reflect framing individual risk factors to a greater extent, as NCD issues are amplified in individual’s psychological burden to guide health authorities to operate specifically. The news articles on the framing strategies of NCDs in mainland China and the neighboring areas differ in several respects. Besides editorial consideration, these differences may also imply a broader healthcare environment for different strategies in priority-setting. Newspapers in mainland China reflect that political leaders actively give attention to an issue and back up that attention according to the severity of the issue. Moreover, the stories from mainland China paid relatively less attention to cardiovascular diseases than in neighboring regions. It is beyond the scope of this study to examine why the issues arose. However, it seems to be plausible that a newspaper owned by the ruling party reflects the preference of the leadership. In other words, the low attention paid to the diseases might indicate that the elite would like it best if not much were being said about these conditions.

### 4.1. Practical Implications

There were substantial variations across mainland China and its three neighboring settings. The baseline information on the magnitude of the problem of risk assessments provided by this study can help health policymakers to set up interventions addressing the Chinese NCDs epidemic. Additionally, this study focused on the coverage of NCDs in online press by setting the pathway into a new era of datamining method to research practices [54,56,57]. Adopting automated content analysis, the data analysis happened simultaneously with data collection. The framing of NCD diseases and causes will have repercussions in the presentation of consequences and the actions taken. Thus, researchers are able to track real-time concerns on diseases and test the feasibility of automated coding.

Framing analyses differ sharply in their procedures, emphases, and assumptions from sociological or psychological approach [32,58,59]. For instance, the sociological portrayal of Alzheimer’s disease and of persons with Alzheimer’s disease were different in the Arabic vs. Hebrew online newspapers; Arabic newspapers were found to concentrate more on objective, health-related and expert-based information [60]. Previous systematic review research suggests that public health problems become amenable to broad policy solutions when those problems can be reframed in systemic terms [8,18,59]. Findings across these areas alert us how the media shape public discourse by setting particular diseases and focusing public interest on explicit risk assessments. 

In practice, the public debate on NCDs will turn on the question of who or what is responsible for causing and curing this emerging epidemic. The findings suggest that a vigorous frame contest is currently under way between arguments emphasizing personal responsibility for health and arguments emphasizing the governmental responsibility and social costs. Specifically, the risk factor of poor diet received very low values of media attention in mainland China (0.2%), HK (0.0%) and Macao (0.1%); the risk factor of lacking of exercise also received scant media attention in Taiwan’s newspapers (0.1%). The evidence shows that media coverage can operate to limit understanding the importance of diet and exercise. Notably, these findings defy expectations of common knowledge and professional reports from the WHO [5,6] and local government reports [14]; these reports strongly advised that improvement of poor diet and insufficient exercise would reduce the prevalence of NCDs. Besides supporting local government and other stakeholders’ engagement, it is imperative to have sufficient coverage in the news media for achieving a better informed public that understands NCDs better.

### 4.2. Limitations

Following the development and increasing use of framing hypothesis [18,31,38], this study is neither straightforward nor unquestioned. Several limitations are noteworthy: First, it is noted that several illnesses of NCDs are closely associated with other risk factors such as diabetes is implicated in the development of obesity, and both overlap with cardiovascular diseases; colorectal cancer is related to obesity, physical inactivity, and eating habits; and some cases of breast cancer and colorectal cancer are highly related to family gene mutation or variation [1,6,15]. However, limited illnesses and risks on the codebook are meant to be supportive and not exhaustive. Secondly, the framing effect has been criticized as disregarding sensitive content and the fine differentiations language offers [18]. It is important to note that the framing idea for this research design lives and dies with the ability to identify implicit causal assertions. By consisting of synonyms for diseases along with risks and consequences assessment, the framing effect was of only small size with either nonsignificant or in the direction opposite to prediction.

## 5. Conclusions

An overall growing interest in NCDs coverage was found for Chinese newspapers, which is important for the successful propagation of information on chronic diseases as a socially and scientifically credible threat to public health. The study was tasked to deal with a new type of metadata that required specific methods to fill the knowledge gap of diseases and risk assessment. As there are few analyses examining NCDs issues in online media, this study provided a description of news information available to the public, shared evidence by identifying research priorities, and discussed how framing of NCDs should be understood. This study considers framing as a research paradigm because framing involves a process of selection in which some aspects of reality are highlighted and others are neglected. We do not see the frame directly but infer its presence by its characteristic expressions in the news. In this manner, automatic content analysis has been used as a key method for investigating disease coverage, risk interpretation, and consequences of the NCDs described.

Empirical research on public health communication is now facing fundamental challenges caused by the rapid diffusion and dynamic development of internet-based platforms. Therefore, this study employs a broader view of interdisciplinary research of computational communication and public health on the drivers of health. It is concluded that Chinese people are exposed to online news which often focus on two NCDs with an emphasis on both non-behavioral and individual risks but less on societal costs. The results evidenced how media frames of NCD topics shape the way in which diseases are selected and interpreted. Although there are scarce works addressing the way media frames potentially shape public consciousness [19,22], a settled rule is suggested for operationalizing the approach to cope with diversified research emphases and procedures. Future research building on our findings can further examine whether news framing affects the way lay people perceive the causes of NCDs and how to prevent them.

## Figures and Tables

**Table 1:** A comparison of NCDs coverage on seven diseases categories in mainland China, Taiwan, Hong Kong, and Macao between 2010–2019.

NCDs	Mainland China	Taiwan	Hong Kong	Macao	All
	(*n* = 32,633)	(*n* = 78,583)	(*n* = 35,818)	(*n* = 5776)	(*n* = 152,810)
	%	%	%	%	%
Metabolic	40.8	34.3	35.4	41.3	36.2
Cardiovascular	16.6	27.3	30.0	24.0	25.5
Cancer	19.9	15.2	18.7	15.7	17.1
Autoimmune	18.1	20.4	12.8	14.4	17.9
Musculoskeletal	4.1	2.4	1.9	3.2	2.7
Neurological decline	0.3	0.3	1.3	0.5	0.5
Chronic respiratory	0.2	0.1	0.0	0.8	0.1

**Table 2:** Risk assessment of NCDs coverage in mainland China and Taiwan, Hong Kong, and Macao between 2010–2019.

Risk	Mainland China	Taiwan	Hong Kong	Macao	All
	(*n* = 14,817)	(*n* = 20,971)	(*n* = 9055)	(*n* = 1743)	(*n* = 46,586)
	%	%	%	%	%
Stress burden	25.1	29.4	29.9	28.2	28.1
Genetic predisposition	24.1	25.4	26.3	18.6	24.9
Tobacco use	13.6	17.6	17.3	19.6	16.4
Pollution damage	12.8	14.1	10.3	13.5	12.9
Alcohol consumption	9.7	8.1	11.0	9.8	9.2
Poverty	8.8	3.4	3.5	6.5	5.3
Lack of exercise	4.1	0.1	0.7	2.2	1.6
Drugs use	1.3	1.5	0.9	1.1	1.3
Personal and family situation	0.1	0.2	0.2	0.3	0.2
Poor diet	0.2	0.2	0.0	0.1	0.2
Social and economic system	0.1	0.0	0.1	0.2	0.1

**Table 3:** Cost consequences of NCDs in newspaper coverage in mainland China, Taiwan, Hong Kong, and Macao between 2010–2019.

Consequence	Mainland China	Taiwan	Hong Kong	Macao	All
	(*n* = 1166)	(*n* = 795)	(*n* = 265)	(*n* = 46)	(*n* = 2272)
	%	%	%	%	%
Societal costs	71.6	68.8	54.3	37.0	67.9
Loss of wealth	12.3	5.5	14.7	13.0	10.3
Family burden	7.7	12.6	18.1	13.0	10.7
Premature death	4.3	5.3	6.4	19.6	5.2
Loss of quality life	3.8	0.4	0.0	13.0	2.3
Other diseases	0.3	5.8	6.0	4.3	2.9
Employer costs	0.0	1.6	0.4	0.0	0.6

## Appendix A

### Target Words of Diseases and Risk Assessment, and Consequences of NCDs

(a) non-communicable diseases (非傳染性疾病)
(b) cardiovascular (心血管)
(c) cardiovascular diseases (心血管疾病)
(d) hypertension (高血壓)
(e) chronic diseases (慢性病)
(f) respiratory tract (呼吸管道)
(g) cancer (癌症)
(h) lung cancer (肺癌)
(i) diabetes (糖尿病)
(j) smoking (抽菸) (食菸) (吸菸)
(k) unhealthy diet (不健康飲食)
(l) too little exercise (少運動)
(m) alcohol consumption (喝酒; 飲酒)
(n) brain stroke (中風)
(o) heart attack (心臟病)

## Appendix B

### Categories of Illnesses 疾病类型

A. Cancer (癌症)
B. Cardiovascular diseases (心血管疾病)
C. Metabolic diseases (代谢性疾病)
D. Auto-immune diseases (自身免疫疾病)
E. Neurological decline (神经衰弱)
F. Musculoskeletal (肌肉骨骼系统)
G. Chronic respiratory diseases (慢性呼吸)

#### A. Cancer (癌症)

(a) Lung cancer (肺癌)
(b) brain cancer (腦癌)
(c) skin cancer (皮膚癌)
(d) blood cancer, leukemia (血癌，白血病)
(e) liver cancer (肝癌)
(f) colorectal cancer (結腸直腸癌)
(g) pancreas cancer (胰腺癌)
(h) breast cancer (乳腺癌)
(i) ovarian cancer (卵巢癌)
(j) uterine cancer (子宮癌)
(k) prostate cancer (前列腺癌)
(l) testicular cancer (睾丸癌)
(m) other (其他癌症)

#### B. Cardiovascular Diseases (心血管疾病)

(a) stroke (中風)
(b) cardiovascular infarction, heart attack (心血管梗塞、心臟病發作)

#### C. Metabolic Diseases (代謝性疾病)

(a) chronic kidney disease (慢性腎臟病)
(b) diabetes (糖尿病)
(c) overweight, adiposities, obesity (超重/過重、肥胖、肥胖)

#### D. Auto-immune Diseases (自身免疫疾病)

(a) allergies (過敏)

#### E. Neurological Decline (神經衰弱)

(a) Alzheimer’s disease (阿爾茨海默氏病) (腦退化症)
(b) Parkinson’s disease (帕金森氏病)
(c) multiple sclerosis (多發性硬化症)

#### F. Musculoskeletal (肌肉骨骼系統)

(a) osteoporosis (骨質疏鬆)
(b) rheumatological arthritis (風濕性關節炎)
(c) fibromyalgia (纖維肌痛)

### Risk Assessment (風險)

(a) Poor diet (不當飲食; 不良飲食)
(b) Lack of exercise (缺乏鍛煉; 缺少運動)
(c) Consumption of alcohol (飲酒; 喝酒; 酗酒)
(d) Tobacco use (吸煙; 食菸; 抽菸)
(e) Drugs other than alcohol or tobacco (毒品; 除了酒精和煙草外的藥物)
(f) Pollution, environmental damages (污染; 環境破壞)
(g) Poverty (貧困; 貧窮)
(h) Psychological burden, stress (心理負擔; 壓力)
(i) Personal and family situation (個人和家庭狀況; 個人居家情況)
(j) Personality traits, psychological predispositions (個人特質; 心理誘因)
(k) Social and economic system (社會和經濟制度)
(l) Genetic predisposition (遺傳)
(m) Others (其他)

### Consequences (結果)

(a) Premature death (過早死亡)
(b) Loss of wealth, financial independence (經濟損失; 財富減少; 經濟失去依靠)
(c) Loss of quality of life (生活品質下降)
(d) Family burden (家庭負擔)
(e) Societal Costs of health system, insurance, public budgets (健康系統; 保險或公共花費; 社會成本)
(f) Costs to employer, patient’s enterprise (企業用人成本; 雇主花費）
(g) Other diseases (其他疾病)
(h) Others (其他)
